# The Influence of Environmental and Genetic Factors and Training Background on the Welfare of Herding Dogs

**DOI:** 10.3390/ani16040607

**Published:** 2026-02-14

**Authors:** Bogumiła Pilarczyk, Renata Pilarczyk, Małgorzata Bąkowska, Agnieszka Tomza-Marciniak, Ewa Kwita, Jan Udała

**Affiliations:** 1 Department of Animal Reproduction Biotechnology and Environmental Hygiene, Faculty of Biotechnology and Animal Science, West Pomeranian University of Technology in Szczecin, Klemensa Janickiego 29, 71-270 Szczecin, Poland; bogumila.pilarczyk@zut.edu.pl (B.P.); agnieszka.tomza-marciniak@zut.edu.pl (A.T.-M.); ewa.kwita@zut.edu.pl (E.K.); jan.udala@zut.edu.pl (J.U.); 2 Department of Biostatistics, Bioinformatics and Animal Research Methods, Faculty of Biotechnology and Animal Science, West Pomeranian University of Technology in Szczecin, Klemensa Janickiego 29, 71-270 Szczecin, Poland; renata.pilarczyk@zut.edu.pl

**Keywords:** genetic predisposition, herding behaviour, herding dogs, positive training, stress in dogs, welfare

## Abstract

Herding dogs play an important role in the management of herds of sheep and cattle, and their effectiveness depends on various genetic and environmental factors, and the choice of training method. The aim of this study is to explain how these factors determine the effectiveness of herding work, the level of stress and psychological comfort experienced by the dogs, as well as their physical health. The study also examines the choice of training method, the influence of socialisation and relationship with the handler, as well as the living conditions. Information on the welfare of herding dogs and the factors that influence this welfare were obtained based on a search of PubMed, Web of Science, Google Scholar and Scopus using defined keywords. Research indicates that positive reinforcement, early socialisation and trust-building with the handler increase performance, reduce behaviour indicative of any stress the dogs may be experiencing, and improve psychological wellbeing. Selective breeding has developed herding predispositions, including herding instinct and cognitive abilities, at the expense of predatory instinct. Understanding the genetic and environmental factors associated with wellbeing, and using ethical training methods benefits both dogs and livestock by allowing herding dogs to fully realise their natural behaviours.

## Simple Summary

The factors influencing the performance and welfare of herding dogs remain relatively unknown. Therefore, the aim of this review is to systematise the current understanding of the genetic predispositions, training methods, handler relationships and environmental factors believed to influence herding dog welfare and behaviour. The review includes 64 relevant publications, and takes into account the different dog breeds, their behaviour when working with a flock of sheep and the impact of training methods on stress and behavioural problems. It was found that welfare is strongly influenced by genetic predisposition and properly conducted training, socialisation and bonding with the handler; in addition, the efficiency of the herding dogs, and the stress levels of both dogs and livestock, can be improved through positive reinforcement, early socialisation and appropriate working conditions. The review also indicates the need for further research into the interaction between genetic and environmental factors in shaping herding dog behaviour.

## 1. Introduction

Shepherd dogs are among the most widely used working dogs in the world [1]. Generally speaking, they serve as herding dogs, which help direct and maintain the cohesion of the herd and as livestock guardian dogs (LGDs), which specialise in protecting the herd from predators. Herding dogs control the movement of the livestock and keep the animals together, using a modified predatory instinct to observe and direct the herd without causing harm [2]. In accordance with the European Parliament [3] definition, a “Herding dog” is an animal that is kept or trained to manage, move or control livestock in agricultural, pastoral and transport conditions. This definition emphasises both the utilitarian function of herding dogs and the need for them to have appropriate behavioural predispositions and cooperate with humans.

By facilitating the work of herders and improving production efficiency, herding dogs play a crucial role in managing cattle and sheep flocks. The herding behaviour of herding dogs is determined by both genetic and environmental factors, with their predisposition to herd being strongly influenced by hereditary traits [4].

However, to best take advantage of their natural predisposition, a dog requires proper training, suitable breeding conditions and commitment by the handler [5,6,7]. Genetically, it is important to select parents with the desired behavioural predispositions, temperament and health to provide their pups with the appropriate potential for herding work. Environmentally, the pups should be provided with suitable breeding conditions and early socialisation, such as access to physical stimuli, and contact with other dogs and people; they should also experience a safe, stimulating environment that promotes the development of herding instincts and cognitive abilities.

The behaviour of herding dogs derives from many years of selective breeding, which has shaped both their cognitive abilities and the instincts necessary for working with animals [8,9]. Such dogs show a unique predisposition that allows them to cooperate with humans, respond precisely to the handler’s signals, and control herd animals with minimal aggression [10].

Modern genetic research indicates that herding dog behaviour is governed by the effects of multiple genes rather than a single “herding gene” [11]. Genome-wide association studies (GWAS) encompassing 268 complete genome sequences from 130 breeds have identified a number of genes associated with herding behaviour, predatory instinct, temperament, and trainability. For example, 44 loci on five chromosomes, associated with neuronal genes such as *THOC1*, *ASIC2*, *MSRB3*, *LLPH*, *RFX8* and *CHL1*, are believed to influence herding behaviours. Some of them, such as *MSRB3* and *CHL1*, are associated with the control of anxiety and fear. Such control is an important characteristic in herding work, where the dog has to limit its killing instinct [11]. The presence of genes such as *JAK2*, *MEIS1* and *LRRTM4*, associated with predatory instincts, and *ACSS3*, associated with temperament, indicates that the behavioural predispositions of herding dogs have a strong genetic basis [11].

While genetic factors have a considerable influence on the natural abilities of herding dogs, appropriate training and early socialisation are also needed to fully exploit their potential [10,12]. A well-planned training environment not only supports the development of cognitive skills but also improves well-being by reducing stress and frustration [13].

Success in herding—defined as achieving expected results in inter alia herding—obedience, and cooperation with the handler, depends on environmental conditions, the age of acquisition of the dog, its maintenance conditions and the quality of interaction with humans. Arnott et al. [5] reported higher success rates among herding dogs acquired at a younger age and kept in group runs or pens, and which participate in work trials. Performance was found to be increased by positive reinforcement, regular exercise and a high level of conscientiousness on the part of the owner and decreased by the use of electric collars. The breed of the dog also mattered, with cattle dog cross hybrids achieving lower success rates than other breeds. Also, dogs perceived by their owners as companions worked more effectively than those treated only as a resource to be worked. Hence, the welfare of herding dogs is strongly dependent on an interplay between appropriate factors, i.e., maintenance conditions, training and owner involvement.

The stress experienced when working with sheep, and humans, also has a considerable influence on the welfare of herding dogs, which are often exposed to situations requiring quick reactions, focus and decision-making under pressure, which can increase stress levels. Rooney et al. [14] note that dogs under higher levels of stress perform worse during training. Stress can impair various cognitive abilities, such as concentration, memory and problem-solving skills, leading to slower scent recognition, erroneous decision-making, faster fatigue and decreased motivation.

Stress levels can be significantly reduced by properly conducted training based on positive reinforcement and trust-building between dog and handler, resulting in clear gains in performance [15]. Dogs that have a stable bond with their handler learn new tasks faster, respond better to commands and are less likely to be stressed in difficult situations [16,17]. In contrast, inappropriate training methods based on punishment and pressure can lower effectiveness by reducing trust and increasing tension [18]. It is therefore crucial to take an appropriate approach to training, socialisation and building a bond between dog and handler, not only to ensure the welfare of the dogs, but also to achieve high standards of work on livestock farms.

Ensuring the welfare of herding dogs, and taking into account their behavioural, genetic and environmental needs, not only improves their emotional well-being and work efficiency, but also, most importantly, helps them maintain good physical condition.

However, it is also difficult to accurately assess animal welfare in general, and mistakes can lead to unnecessary suffering. As such, there remains considerable interest in identifying the most reliable and credible methods to assess the welfare of dogs [15].

The aim of this article is to provide a comprehensive analysis of the genetic, behavioural, environmental and training factors influencing the welfare of herding dogs. It examines the influence of genetic predisposition, training method, socialisation, relationship with the handler and maintenance conditions on efficiency, stress levels, psychological comfort and physical health in herding dogs. The article also identifies practical implications for improving the welfare of dogs and the effectiveness of their work with the herd.

## 2. Materials and Methods

### 2.1. Study Design

This study was conducted as a systematic review in accordance with PRISMA 2020 guidelines. Its aim was to identify, evaluate and synthesise available evidence on the impact of genetic, environmental and training-related factors on the welfare of herding dogs.

### 2.2. Search Strategy

The review corpus was created by searching PubMed, Web of Science, Google Scholar and Scopus databases using the following keywords: “herding dogs”, “working dogs”, “dog welfare”, “animal welfare”, “herding behavior”, “herding dog breeds”, “working dog performance”, “positive reinforcement training”, “dog training methods”, “puppy socialization”, “stress in working dogs”, “behavioral problems in dogs”, “dog-handler relationship”, “human-dog interaction”, “canine cognitive abilities”, “genetic predispositions of herding dogs”, “sheepdog training”, “musculoskeletal injuries in working dogs”, “dog welfare assessment methods”.

The search included logical operators (AND/OR) to broaden or narrow the results. No restrictions were imposed on the year of publication. It only included articles in English and available in full text. Sample queries in databases:

- PubMed:

“Herding Dogs” OR “working dogs” OR “sheepdog” OR “herding breeds” AND “dog welfare” OR “animal welfare” OR “stress in dogs” OR “behavioral problems” AND “positive reinforcement training” OR “dog training methods” OR “human-dog interaction”

- Scopus:

“herding dogs” OR “working dogs” OR “sheepdog” OR “herding breeds” AND “dog welfare” OR “stress in dogs” OR “behavioral problems” OR “human-dog interaction” AND “positive reinforcement” OR “training methods”

- Web of Science:

“herding dogs” OR “working dogs” OR “sheepdog” OR “herding breeds” AND “dog welfare” OR “stress in dogs” OR “behavioral problems” OR “human-dog interaction” AND “positive reinforcement” OR “dog training”

- Google Scholar:

“herding dogs” OR “working dogs” OR “sheepdog” AND “dog welfare” OR “stress in dogs” OR “behavioral problems” OR “human-dog interaction” AND “positive reinforcement” OR “dog training” (Table 1).

### 2.3. Inclusion and Exclusion Criteria

- Inclusion criteria

Studies were included if:

- They concerned the welfare of herding or working dogs in various training and environmental contexts;
- They analysed the impact of training methods, socialisation and human–dog interactions on stress, behaviour and performance;
- They contained information on behavioural problems and the use of positive reinforcement methods to improve welfare;
- They presented results on genetic predispositions, breed differences and environmental factors affecting herding behaviour and overall welfare.

- Exclusion criteria

Studies were excluded if:

- They did not involve herding or working dogs;
- They were duplicates;
- The full text was missing;
- They did not meet the inclusion criteria.

### 2.4. Study Selection

The search identified 808 publications. This number was reduced to 143 by removing duplicates and unrelated material. After evaluating the full texts, 79 studies were excluded due to lack of full text or failure to meet the inclusion criteria. Therefore, 64 publications were included in the qualitative synthesis.

### 2.5. Data Collection

The following information was extracted from the publication:

- Authors and year of publication;
- Study design and objectives;
- Breed and population characteristics;
- Training methods and management systems used;
- Environmental and genetic factors studied;
- Studied welfare indicators (*viz.* behavioural, physiological and health);
- Key findings regarding stress, welfare and performance.

### 2.6. Data Synthesis

As the included studies were characterised by considerable heterogeneity with regard to research projects, populations and outcome measurement methods, no quantitative analysis (i.e., meta-analysis) was performed. Instead, a narrative approach was used, in which the data were grouped thematically and presented in structured subsections. Its aim was to provide a comprehensive description of the genetic, behavioural, environmental and training-related factors affecting the welfare of herding dogs.

### 2.7. PRISMA Diagram

**Figure 1:**
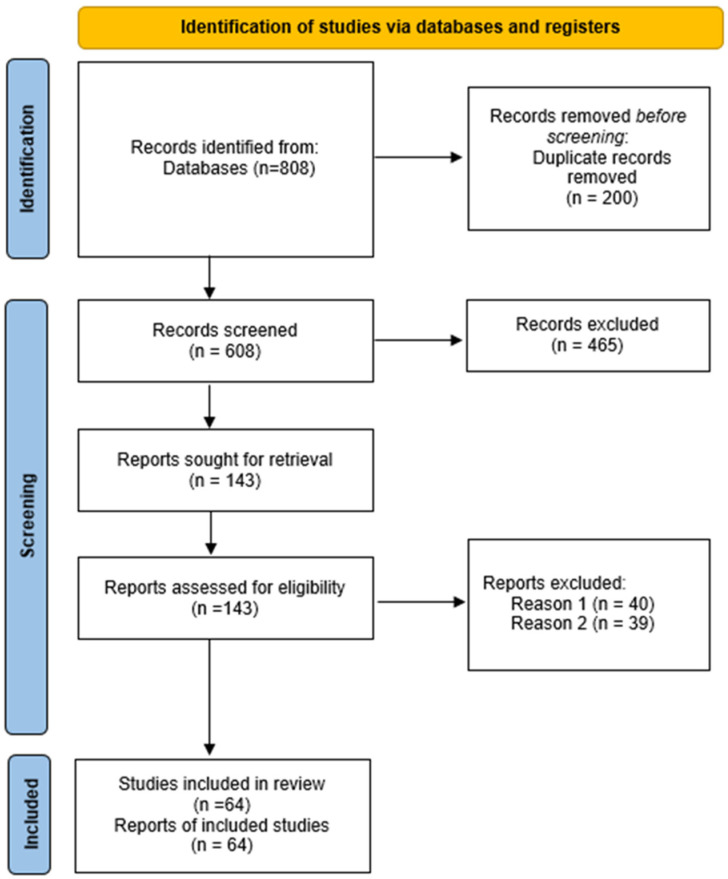
The process by which publications were identified and selected for the literature review based on Page et al. [19].

The process by which the studies were identified, selected, and included in the study is presented in Figure 1. The procedure was performed in accordance with PRISMA 2020 guidelines.

## 3. Determinants of the Welfare of Herding Dogs

Our findings indicate that the welfare of herding dogs does not depend on a single factor, but on the interaction of several important elements. The included studies primarily address four main issues. The first concerns matching a dog’s genetic predisposition to the requirements of herding work. The second involves the quality of training and the level of cooperation between the dog and the handler, which affect both the effectiveness of the dog’s work and its stress levels. The third issue emphasises the importance of living conditions and work organisation for physical and mental well-being. Finally, they analyse the effects of grazing practices on both the dogs and the livestock under their control.

The effectiveness of a herding dog is determined by both their genetic characteristics and their environment. However, individual breeds differ in their responses to stimuli, their motivation to cooperate with humans and their natural herding behaviour [20,21,22]. For example, more cooperative dogs are more likely to follow human cues than more independent breeds. These traits may also be influenced by how the dog is treated and by their physical characteristics, such as size or eye placement [23].

Herding skills are important not only for the dogs themselves, from the perspective of their utility, but also for the wellbeing of the animals in their care. Herding dogs with a good genetic pedigree and proper training are able to control a flock in a less stressful manner, which benefits both dog and livestock [24]. Improperly matched dogs or lack of proper training, on the other hand, can increase the level of stress and danger to animals and dogs [25].

## 4. Genes and Behaviour in Herding Dogs

The behaviour of herding dogs has been shaped by many years of breeding selection, which has influenced both their cognitive abilities and instincts related to working with the herd. However, while genetic predispositions play a key role in shaping working efficiency, correct training and socialisation are needed to fully exploit this potential.

### 4.1. Genetic Selection and Behavioural Predisposition

Generations of shepherd dogs have been selected for the qualities needed to work with sheep, such as precise movements, high motivation to perform difficult tasks and the ability to recognise handler and animal signals [8,9]. The characteristic behaviours of these breeds, such as watching, stalking animals and chasing them without harming them, are the result of multi-generational selection. However, fully exploiting this genetic predisposition requires properly targeted training, combining obedience exercises with tasks to stimulate cognitive abilities, thus exemplifying the synergy between genotype and environment [10,12].

Selective breeding has focused on developing traits such as the instinct to herd, the willingness to cooperate with humans, responding quickly to handler signals and controlling the herd with minimal aggression, as well as a strong focus and controlled excitability. Genetic studies have shown that many of these traits have a strong hereditary basis, while some, such as the ability to concentrate and controlled excitability, also depend on early socialisation and training [8,9,10,11] (Table 2).

Research, however, indicates that no single “herding gene” or “training gene” exists, but rather, that multiple genes responsible for social interaction and cognitive function play a role in the selection of herding dogs [13]. Indeed, an analysis of the genomes of 12 pastoral breeds and 91 non-pastoral breeds found the selection criteria to encompass hundreds of genes, some of which were linked to cognitive functions. In particular, more than eight genes strongly associated with cognitive functions were identified in Border Collies, including *EPHB1*, responsible for spatial memory and the regulation of motor patterns. The fact that herding breeds carry a number of *EPHB1* variants suggests they play a role in the decision-making processes necessary for effective sheep handling.

A study of 130 dog breeds found 44 loci on five chromosomes (*CFA1*, *CFA6*, *CFA7*, *CFA9*, *CFA10*, *CFA17*, *CFA20*) to be significantly associated with herding behaviour [11]. Various genes appear to be associated with nervous system function and behavioural regulation, including *THOC1*, *ASIC2*, *MSRB3*, *LLPH*, *RFX8*, *CHL1*, *JAK2*, *MEIS1*, *LRRTM4* and *ACSS3*. Some of these (e.g., *MSRB3* and *CHL1*) have previously been linked to fear in dogs, which may affect instinct control when working with animals.

### 4.2. Genes and Predatory Instinct

A comparison of herding and hunting breeds found that some genes associated with predatory behaviour (*JAK2*, *MEIS1*, *LRRTM4*) can be modified to reduce the killing instinct. The JAK2 gene is involved in synaptic plasticity and memory, MEIS1 affects the impulsive pursuit reflex and activity, while *LRRTM4* promotes the development of excitatory synapses and is linked to predatory aggression [11]. This instinct has been modified over generations of breeding herding dogs, suppressing the urge to kill prey in the final stage of the hunt [11].

Understanding genetic predispositions and providing the right environment and training allow dogs to fully realise their natural behaviour; as such, they minimise stress and frustration and are important components in ensuring wellbeing [13]. Beaver [10] report that some traits, such as the ability to cooperate in a group or to adapt quickly to new tasks, are partly heritable, confirming the role of genetic selection in the formation of herding skills.

### 4.3. The Genetic Basis of Behaviour and Predisposition in Herding Dogs

Herding dogs are bred primarily for desirable behaviours such as target concentration, following, and chasing, which improves their effectiveness in tasks that require them to respond to human movements [20]. The Border Collie, for example, is more sensitive to moving stimuli and has a greater motivation to chase than guard dogs or some hunting dogs, making it better in target-pointing tasks [25]. Udell et al. [25] attribute the underperformance demonstrated by some breeds, such as the Anatolian shepherd, to innate behavioural inhibitions rather than a lack of cognitive ability: after additional training, these dogs quickly mastered their tasks, demonstrating that both innate predisposition and experience influence performance in cognitive tasks. Indeed, differences between breeds often reflect their natural behaviour and previous experience, rather than cognitive ability alone, which is important in the selection and training of herding dogs.

The Border Collie has a highly developed herding instinct that naturally drives the dog to direct herds. This instinct is believed to be rooted in its genes, and to stem from the organisation of the neural networks of the brain. The breed is also characterised by exceptional energy and concentration, which is linked to variants of the genes responsible for nerve impulse conduction (i.e., axon guidance). Thanks to these genetic predispositions, these dogs show high motivation and focus in tasks requiring intense activity and attention [26].

Australian Shepherds are distinguished by their high energy and diligence, which have been enhanced by genetic selection favouring motor skills and cognitive functions. These dogs also respond very quickly to visual and auditory signals, which has been attributed to neural genes associated with rapid stimulus processing; this translates into efficient task performance and high activity [11,13].

The Shetland Sheepdog is able to maintain concentration for long periods of time and cooperates well with its owner. Again, these traits are thought to be genetically determined: the rapid learning of new commands and efficient performance of tasks requiring precision and discipline by the dog have been associated with changes in the genes responsible for the functioning of nerve receptors and signal conduction in the brain [13].

The Belgian Malinois, however, is characterised by a high degree of independence and physical resilience. It is able to assess situations and make decisions without constant human supervision, which may derive from selective changes in genes related to cognitive functions, while its high endurance may be ensured by genes regulating metabolism and neuronal activity. As such, this breed is particularly suitable for tasks requiring both physical and intellectual effort. Despite this, proper training is necessary to fully realise its potential [13].

The long-haired Collie is characterised by a gentle and predictable temperament, which is the result of genetic selection favouring cooperation with humans. It is believed that the Collie’s temperament is influenced by the genes controlling neurotransmission and emotional responses. Indeed, the breed is calmer, more obedient, and easier to interact with on a daily basis than the more energetic and independent herding breeds [26] (Table 3).

## 5. Behavioural Differences Between Sex and Breed

Research has shown that working breeds such as herding dogs exhibit distinct behavioural differences with non-working breeds. They are more receptive to training, learn new commands more quickly and are capable of performing complex tasks when working with a handler. In addition, these dogs show greater interest in interacting with humans, which facilitates cooperation when working in a herd; they also more resistant to anxiety, allowing them to function effectively in the dynamic and stressful conditions associated with herding work [27].

Working breeds constitute a coherent behavioural group separate from non-working breeds. The former are characterised by predictable patterns of response to training and human signals, while the latter show greater behavioural variability, with less consistent responses that are more dependent on individual characteristics (Table 4) [9,27].

Such behavioural differences are consistent with the historical purpose of herding dogs, which favoured agility, precision, cooperation and stress resistance. Low levels of fearfulness and aggression are needed to control herds without harming the animals, while high trainability and interest in humans promote effective cooperation with the handler [8,9].

Males tend to exhibit greater aggression and boldness; they are better suited to protecting the herd and quickly adapting to changing terrain, but they are less inclined to cooperate with humans and have less visual focus on individual stimuli. In contrast, females are less aggressive, more cooperative, better focused on individual stimuli and systematic in their work (Table 5) [28].

## 6. Impact of Training Methods on the Welfare of the Dog

Understanding the factors that influence dog welfare is one of the most important aspects of research into human–animal relationships, and the method of training is a key factor influencing animal behaviour and psychological comfort. Studies have identified an observed association between the choice of training method and the occurrence of behavioural problems, which are a potential indicator of reduced welfare [29]. These findings are particularly relevant for herding dogs. As these breeds are characterised by high cognitive abilities, emotional sensitivity and a strong instinct to cooperate with humans, they require appropriately selected training strategies. A study by Blackwell et al. [29], examined a broad population of dogs of different breeds, of which 18% were herding dogs. The findings identified a link between the type of training method and the occurrence of undesirable behaviours such as attention seeking, anxiety (avoidance) and aggression. Dogs trained exclusively using positive reinforcement methods, such as treats, praise or play, showed the lowest incidence of undesirable behaviour, while those trained using aversive methods, such as shouting, leash jerking or physical punishment, were more likely to exhibit behavioural problems. In addition, the use of positive punishment correlated with a higher chance of negative reactions, as reported by 72% of owners. Hence, training methods have a measurable impact on dog behaviour and well-being, regardless of breed.

Hence, the innate predisposition of herding dogs, i.e., their high intelligence, readiness to work and willingness to cooperate, does not protect them from the negative effects of inappropriate training methods.

Another observational study by Schalke et al. [30] on the impact of electric collars on dogs in everyday situations found that the use of such aversive tools can cause symptoms of stress and foster undesirable behaviours in dogs. In contrast to Blackwell et al. [29], who surveyed owners of pet dogs, Schalke et al. [30] based their research on direct observations of working dogs in training conditions. Their findings highlight the potential risks to animal welfare associated with the use of aversive methods such as electric collars.

The use of punishment in the training process not only reduces the effectiveness of learning but also weakens the human–animal relationship. Dogs may perceive the owner as a source of threat, which creates fear and uncertainty and makes it more difficult to build trust [29,31]. As a consequence, these animals are more likely to react with fear or aggression to a simple reprimand [32]. This indicates that methods based on pressure and coercion violate the dog’s sense of security.

Marschark and Baenninger [33] propose that while positive reinforcement plays an important role in training herding dogs, it is insufficient in itself to effectively control behaviour based on strong instincts. In such cases, blocking access to sheep and using other forms of gentle punishment may be necessary to help form correct working patterns. The authors propose combining positive and negative forms of reinforcement with selective use of punishment to both reward desirable behaviour and effectively reduce undesirable instinctive responses (Table 6).

The study also found socialisation to have a significant effect on the behaviour of adult dogs, and that participation in puppy socialisation classes allowed for early positive experiences with other animals and people. Dogs participating in this type of activity as puppies were less likely to show adverse reactions to strange dogs as adults, confirming that proper socialisation plays a key role in developing emotionally stable and balanced adult dogs [7,29]. Inconsistency in communication, such as the use of both rewards and punishment by the owner, led to increased frustration and learning problems on the part of the dog. This phenomenon is particularly relevant for herding dogs, which are extremely sensitive to subtle handler cues and require precise guidance while training [33]. Indeed, consistency, clarity of instruction and building a trusting relationship are the cornerstones of effective and ethical training.

Due to their high intelligence, good cognitive abilities and strong instinct to work with the herd, herding dogs require a special approach during training. Taking a suitable approach not only improves cooperation with the handler, but also the welfare of the herd by reducing stress and the risk of injury [24]. For example, the *stalking posture* observed in the Border Collie, an innate predisposition which resembles the behaviour of a predator, allows the dog to direct the movement of the pack more effectively. Nevertheless, the ability of the dog to use this posture correctly relies on both appropriate training and the handler’s ability [34].

Previous methods of working with herding dogs have often relied on pressure and punishment, but research has found this approach to cause stress and impair concentration, and can even exacerbate aggressive behaviour [31,35,36]. Blackwell et al. [29] indicate that aversive methods lead to a reduction in the welfare of all dogs, regardless of breed. Such reductions were also noted in herding breeds, which constituted the second largest group examined in the study (18%). This is supported in most contemporary research, which clearly indicates that positive reinforcement is the most effective approach when working with herding dogs: rewarded dogs master new skills more quickly, cooperate more readily with their handler and are less likely to exhibit stress reactions [7]. Building a relationship based on reward and mutual trust improves the effectiveness of working with the herd and reduces the number of behavioural problems.

Therefore, it can be concluded that training methods based on positive reinforcement are not only more ethical, but also more effective, especially with herding dogs. Properly conducted training increases the welfare of the dogs, as well as improving their cooperation with humans and bestowing a positive effect on the whole herd.

## 7. The Welfare of Dogs When Working with Sheep

Close contact between a sheepdog and a sheep is a stressful situation for both parties: the sheep react by stamping their feet, and the dogs often lick their muzzles, which is a sign of tension. If the dog maintains the pressure, the sheep may feel threatened and react defensively [37]. The stress felt by the dog can be alleviated through proper preparation and socialising the puppies with sheep early in life. Such measures will also limit predatory behaviour and strengthen its protective instinct [38].

By building its self-confidence and fostering a stable relationship with its handler, it is possible to calm the reactions of the working dog and make them more predictable, in turn reducing the stress placed on the sheep [39]. In contrast, excessive emotional bonds or overprotectiveness can hinder effective work with the flock by causing fear, hyperactivity or anxiety in dogs [40,41].

The relationship with humans has a considerable influence on the well-being and effectiveness of the sheepdog at work. Dogs are sensitive to signals from their handler and can adapt their behaviour to their expectations. Indeed, a close bond based on trust has been found to increase motivation and improve stress management thanks to increased oxytocin levels [42,43,44]. Furthermore, a positive relationship built on positive reinforcement, such as rewarding with treats or praise, has been found to support well-being and accelerate learning [45,46,47].

Incorrect communication, such as intense eye contact or sudden movements, can be stressful for a dog, and ignoring signs such as turning the head away, yawning or licking its lips, makes effective work difficult [48,49]. Nevertheless, early socialisation with people, and familiarisation with sounds and other stimuli, helps dogs cope with difficult conditions, reduces stress and improves their mental well-being [50].

In stressful situations, herding dogs show a strong need for social support, especially by making eye contact and enjoying physical closeness with their owner, reflecting their long history of cooperation with humans [51]. At such moments, they prefer the presence of their owner over a stranger, and their stress responses often synchronise with those of the human, underlining the importance of the dog–human relationship in maintaining well-being [52]. Studies have found the state of mind of the handler to directly affect the behaviour and physiological responses of the dog, including its cortisol levels [51].

### 7.1. Atmospheric Conditions

The combination of physical exertion and long working hours in high temperatures makes working dogs particularly susceptible to overheating and heatstroke, with the risk of hyperthermia depending on *inter alia* workload, environmental conditions, acclimatisation, hydration and the response of the person responsible for the dog [53]. Nevertheless, while all sheepdogs are at risk of hyperthermia while working, individual heat tolerance varies between individuals [54,55,56].

High air humidity increases the risk of overheating even at relatively moderate temperatures by reducing the effectiveness of cooling through evaporation [57]. Unlike many mammals, which cool themselves mainly through sweating, dogs only have sweat glands on the pads of their paws. Therefore, their bodies mainly use panting and increased blood flow in the skin to dissipate excess heat [58]; maintaining the correct body temperature largely depends on environmental conditions, especially during physical exertion: airflow accounts for approximately 60% of total heat loss [59,60].

Heat resistance can be built through gradual acclimatisation and regular activity, which support adaptive cellular processes, including heat shock protein expression [53,61]. However, early recognition of possible overheating can play an important role in ensuring the health and well-being of dogs working at high temperatures [62].

### 7.2. Behavioural and Health Problems in Sheepdogs

It has been estimated that the single greatest reason for removing dogs deemed unsuitable for work was a lack of natural herding ability (54.3%), followed by temperament issues (25.8%), and training difficulties (9.1%). It was also found that only about 10% of dogs were retired for medical reasons or insufficient physical condition [5]. Even so, sheepdogs are at high risk of musculoskeletal injuries, particularly to the wrist and hip, with an appreciable impact on their welfare. Lameness and age are significant risk factors which can shorten their working life [63,64].

A study of working sheepdogs in New Zealand found over 40% to have at least one musculoskeletal abnormality [63]. Of the studied dogs, 57% developed their first abnormality during the observation period, and 68% of this group experienced a second abnormality and 26% experienced a recurring problem in the same location. The most common injury sites were the wrist (16%), hips (12%), toes (11%) and knee joint (8%) [63]. Over a year, 14% of cases were non-traumatic and 12% were traumatic [64].

Musculoskeletal disorders, such as injuries to joints, bones and muscles, were found to significantly limit the ability of dogs to perform work. These problems also resulted in chronic pain and poorer overall well-being. At work, such injuries resulted in reduced performance in protecting herds, difficulty in moving around and limitations in natural behaviours such as running and playing. In addition, chronic pain and discomfort led to stress, frustration and poorer quality of life [63,64].

## 8. Conclusions

The welfare of herding dogs depends on their genetic predisposition, training methods, socialisation and the quality of their relationship with their handler. By providing positive reinforcement, early socialisation and clear communication, the handler can reduce stress, improve mental comfort and work efficiency. Potential behavioural and health problems can also be minimised through suitable breed selection and appropriate working conditions. By creating such a favourable environment, the handler can take full advantage of the dog’s natural herding abilities. Furthermore, by consciously fostering the well-being of the herding dog, based on an awareness of its genetic predispositions, training methods and working conditions, it is also possible to enhance the safety and comfort of the farm animals which it herds (Table 7).

## Figures and Tables

**Table 1:** Logical search table.

Database	Main Keywords	Synonyms/ Extensions	Exclusions	Thematic Aspects
PubMed	herding dogs, working dogs	sheepdog, herding breeds	companion dogs, lab dogs	welfare, stress, behaviour, training, human–dog interaction
Scopus	herding dogs, working dogs	sheepdog, herding breeds	pet dogs	welfare, stress, behaviour, training, human–dog interaction
Web of Science	herding dogs, working dogs	sheepdog, herding breeds	non-working dogs	welfare, stress, behaviour, training, genetics
Google Scholar	herding dogs, working dogs	sheepdog, herding breeds	companion dogs, pet dogs	welfare, stress, behaviour, training, human–dog interaction

**Table 2:** Key genes and their functions according to Shan et al. [11].

Behaviour/Characteristic	Gene	Chromosome	Biological Function	Relationship with Behaviour	State of Evidence
Herding	*THOC1*	CFA7	Presynaptic development, survival of dopamine neurons	Associated with fearlessness traits (boldness) and cognitive function in herding dogs	Confirmed
	*ASIC2*	CFA9	Encoding ion channels (ASICs), involved in the development of the nervous system	Candidate associated with pastoral work; no direct evidence of impact on *fear response*—possible indirect link	Hypothesis
	*MSRB3*	CFA10	Regulation of brain morphology, oxidative stress	Potential effects on neurological function, hearing and stress reactions	High probability
	*LLPH*	CFA10	Neuronal development, synaptic transmission	May affect learning ability and adaptation	Hypothesis
	*RFX8*	CFA10	Schwann cell proliferation, nerve regeneration	May modulate neurological performance, important in the development of the nervous system	Hypothesis
	*CHL1*	CFA20	Nerve cell adhesion, neurite proliferation	Related to intelligence, cognitive functions and emotional responses	High probability
Predation	*JAK2*	CFA1	Synaptic plasticity, regulation of neuronal transmission	Linked to intensity of pursuit and hunting behaviour	Confirmed
	*MEIS1*	CFA10	Specification of neural progenitors	Linked to impulsivity and hyperactivity during pursuit	Confirmed
	*LRRTM4*	CFA17	Development of excitatory synapses	Can influence predatory aggression behaviour	Hypothesis
Temperament	*ACSS3*	CFA15	Regulation of energy metabolism, stress response	Correlates with temperament and emotionality traits	Confirmed

**Table 3:** Genetic basis of behaviour in selected herding dogs.

Breed	Characteristic/ Behaviour	Genetic Basis/Influence	Influence on Behaviour/ Value (Usability)	Source
Border Collie	Herding instinct	Strong genetic basis for herding behaviour linked to brain neural networks	Inclination to automatically control the movement of the herd	[26]
	High concentration and energy	Gene variants associated with nerve impulse conduction (axon guidance)	Extreme focus and motivation to work	[26]
Australian Shepherd	High energy and diligence	Selection of genes facilitating motor skills and cognitive functions	Very strong need to be set activities and tasks	[13]
	Reaction to visual/audio signals	Genes associated with the speed of stimulus processing	Quick response to commands and signals	[11]
Shetland Sheepdog	Focus and obedience	Differences in cognitive genes (receptors and signal transmission)	Long concentration span and fast learning	[13]
Belgian Malinois	Autonomy and independent decision-making	Traces of selective changes in genes associated with social interactions and cognitive functions	Independent, often requiring training	[13]
	Energy/endurance	Modification of neuronal and metabolic genes for supporting extended work periods	Very active, ideal for difficult tasks	[13]
Collie (long-haired)	Mild temperament	Selection of behavioural traits conducive to cooperation with humans	Calmer, more of a “follower” than, for example, a Border Collie	[26]

**Table 4:** Differences in behaviour between working and non-working breeds of dog (based on Eken Asp et al. [27]).

Characteristic	Working Breed (Herding)	Non-Working Breed	Difference
Suitability for training	higher	lower	+10%
Interest in playing with people	more	less	+30%
Timidity	lower	higher	10–60% lower
Aggression	lower (especially in companion dogs)	higher in timid dogs	_
Ease of training in companion dogs	higher	lower	_

**Table 5:** Difference between male and female herding dogs (based on Scandurra et al. [28]).

Characteristic/Behaviour	Males	Females
Aggressiveness and boldness	Higher	Lower
Interactions with humans	Less willing to cooperate	More willing to cooperate
Social play with other dogs	More active in play	Less active
Cognitive flexibility/ navigation	Greater strategic flexibility	Less strategic flexibility
Visual focus	Less focus on individual stimuli	Better focus on individual stimuli

**Table 6:** Comparison of behaviour modification methods for herding dogs based on Marschark and Baenninger [33].

Method	Definition	Example Given in the Study	Effectiveness	Recommendations from the Study
Positive reinforcement	Adding a rewarding stimulus to increase the likelihood of the desired behaviour occurring	Allowing the dog access to sheep or verbal praise	Moderate—giving real permission to access sheep was most effective, while verbal praise had limited impact	Good for learning new commands, but not enough to fully control instinctive behaviour
Negative reinforcement	Removal of aversive stimulus to reinforce desired behaviour	Providing access to sheep after a valid command has been executed	Effective because dogs perceived access to sheep as a reward after being previously restricted	Supports the learning process if applied consistently
Punishment	Adding an aversive stimulus or taking away a reward to undermine undesirable behaviour	Blocking access to sheep or holding the dog	Most commonly used and effective in controlling dogs during instinctive behaviour	In the case of strong instincts, punishment has proven to be an essential part of training

**Table 7:** Factors affecting the welfare of herding dogs (genetics, training, socialisation, environment).

Section/ Factor	Key Findings	Implications for Wellbeing	Recommendations/ Practical Application	Source
Genetic predisposition	Herding traits, cognitive abilities, and weakened predatory instincts are highly heritable; the EPHB1, THOC1, ASIC2, and MSRB3 genes influence herding behaviour and fear responses.	Genetic selection plays a key role in work efficiency and stress reduction; breed differences affect training needs.	Breeding selection based on behavioural traits; selection of dog breed, sex and genetic line for herding work.	[10,11]
Sex differences	Males are bolder and more aggressive; females are more cooperative and focused.	Sex influences task allocation and training strategies.	Adapt training and tasks according to sex.	[28]
Training methods	Positive reinforcement improves learning, reduces stress and behavioural problems; aversive methods increase anxiety, aggression and reduce trust.	Training directly affects wellbeing, relationships with humans and work efficiency.	Use reward methods; incorporate gentle negative reinforcement when necessary; avoid excessive punishment.	[29,33]
Relationship with owner	A strong, trust-based bond improves motivation, reduces stress and increases responsiveness.	Dog welfare and herding performance are dependent on one another.	Build trust, use consistent signals, positive interactions; signs of stress in the handler affect the dog.	[14,16,17]
Socialisation	Early socialisation reduces anxiety, and improves adaptation and cooperation with humans.	Socialisation affects long-term emotional stability and work performance.	Provide socialisation classes for puppies, allow controlled contact with animals and people; consistent communication.	[27,32]
Behavioural and health problems	Musculoskeletal injuries (wrist, hip, knee, fingers) and behavioural problems (lack of herding instinct, anxiety) limit the length and quality of work.	Injuries and behavioural problems reduce well-being and work efficiency.	Regular health checks; early detection of problems; appropriate exercise and weight selection.	[5,28,63,64]
Environmental conditions	High temperatures, humidity and physical exertion increase stress and the risk of overheating.	Physical wellbeing depends on acclimatisation, hydration and safe working conditions.	Monitor environmental conditions; gradual acclimatisation; ensure breaks and hydration.	[29,53,54,55,56,57,58,59,60,61,62]
Article summary	Well-being depends on a synergy between genetics, training, socialisation, relationship with handler and environmental conditions.	Positive reinforcement, selective breeding and good working conditions maximise welfare and efficiency.	Integrated approach: ethical training, breeding selection, early socialisation, environmental control.	

## Data Availability

No new data were created or analysed in this study. Data sharing is not applicable to this article.
